# The influence of long chain polyunsaturate supplementation on docosahexaenoic acid and arachidonic acid in baboon neonate central nervous system

**DOI:** 10.1186/1741-7015-3-11

**Published:** 2005-06-23

**Authors:** Guan-Yeu Diau, Andrea T Hsieh, Eszter A Sarkadi-Nagy, Vasuki Wijendran, Peter W Nathanielsz, J Thomas Brenna

**Affiliations:** 1 Division of Nutritional Sciences, Cornell University, Ithaca, New York, USA; 2 College of Veterinary Medicine, Cornell University, Ithaca, New York USA; 3 Division of Pediatric Surgery, Department of Surgery, Tri-Service General Hospital (TSGH), National Defense Medical Center (NDMC), 325 Chenggung Rd, 2 Sec, Naihu, Taipei 114, Taiwan, Republic of China; 4 Dept of Nutritional Sciences, University of California, Berkeley, CA, USA; 5 Brandeis University, Foster Biomedical Laboratory, Waltham, MA, USA; 6 Dept of Obstetrics and Gynecology, University of Texas Health Science Center, San Antonio, TX, USA

## Abstract

**Background:**

Docosahexaenoic acid (DHA) and arachidonic acid (ARA) are major components of the cerebral cortex and visual system, where they play a critical role in neural development. We quantitatively mapped fatty acids in 26 regions of the four-week-old breastfed baboon CNS, and studied the influence of dietary DHA and ARA supplementation and prematurity on CNS DHA and ARA concentrations.

**Methods:**

Baboons were randomized into a breastfed (B) and four formula-fed groups: term, no DHA/ARA (T-); term, DHA/ARA supplemented (T+); preterm, no DHA/ARA (P-); preterm and DHA/ARA supplemented (P+). At four weeks adjusted age, brains were dissected and total fatty acids analyzed by gas chromatography and mass spectrometry.

**Results:**

DHA and ARA are rich in many more structures than previously reported. They are most concentrated in structures local to the brain stem and diencephalon, particularly the basal ganglia, limbic regions, thalamus and midbrain, and comparatively lower in white matter. Dietary supplementation increased DHA in all structures but had little influence on ARA concentrations. Supplementation restored DHA concentrations to levels of breastfed neonates in all regions except the cerebral cortex and cerebellum. Prematurity *per se *did not exert a strong influence on DHA or ARA concentrations.

**Conclusion:**

1) DHA and ARA are found in high concentration throughout the primate CNS, particularly in gray matter such as basal ganglia; 2) DHA concentrations drop across most CNS structures in neonates consuming formulas with no DHA, but ARA levels are relatively immune to ARA in the diet; 3) supplementation of infant formula is effective at restoring DHA concentration in structures other than the cerebral cortex. These results will be useful as a guide to future investigations of CNS function in the absence of dietary DHA and ARA.

## Background

Docosahexaenoic acid (DHA) is the most unsaturated fatty acid in mammalian tissue. It is found at particularly high concentration in retina [1] and cerebral cortex [2], concentrated mainly in serine and ethanolamine phosphoglycerides [3]. Early observations led to studies showing that low tissue DHA induced by dietary deficiency of n-3 fatty acids results in compromised retinal function as reflected by poor electroretinogram parameters [4-6] and altered cognitive function [7]. Several hypotheses have been proposed to explain the molecular role of DHA, including its high degree of molecular flexibility as a component of membrane phospholipids [8], improvement of G-protein-coupled signaling [9,10], more favorable interaction with integral membrane proteins [11], its ability as a free fatty acid to stabilize the electrical activity of ion channels [12], and, very recently, as a precursor for compounds protective of CNS function during ischemia [13].

The majority of DHA studies have focused on its role in the cerebral cortex. There are few data on DHA concentrations in most deep CNS structures at a gross level. For instance, there are no studies of DHA concentrations in the basal ganglia. These complex structures are involved in a wide array of integrative functions involving motor coordination, integration of visual signals, and psychiatric and personality phenomena [14]. The globus pallidus, caudate nucleus and putamen suffer specific, massive loss of neurons in Huntington's disease, resulting in chorea, dementia and psychiatric disturbances [15]. In Parkinson's disease, elimination of excessive output of the inner segment of the globus pallidus by surgical removal or lesion of the subthalamic nucleus has re-emerged as a treatment for elimination of tremors [16]. The mechanism of neural loss in these diseases remains elusive. Similarly, there are few data on the fatty acid compositions of the limbic regions, the thalamus or the midbrain.

Mammals obtain DHA nutritionally in two forms, either as preformed DHA, found primarily in marine foods, or via the dietary essential polyunsaturated fatty acid (PUFA) α-linolenic acid (18:3n-3), from which DHA is synthesized in various tissues. In vitro data suggest that in the CNS, glia and cerebral endothelial cells, but not neurons, synthesize DHA from 18:3n-3 and other precursor n-3 fatty acids [17]. Once formed, phospholipids containing DHA are rapidly incorporated into neurons, and are concentrated in synaptosomes where they influence neurotransmitter content [18].

There has been intense interest in DHA and its influence over function in the developing CNS. Randomized clinical trials have shown that infant formulas with DHA improve retinal and CNS function compared to formulas containing only 18:3n-3 [19]. All studies also include arachidonic acid (ARA) to prevent any possible negative effects on growth reported in early studies [20]. Virtually all clinical developmental studies in infants have examined visual and cognitive outcomes [19], and are limited to sampling of blood for assessment of tissue responses to supplementation, rather than the target CNS tissues.

Our first purpose in this report is to assess DHA and ARA concentrations in normal, four week old baboons within many CNS structures not previously studied. We also test the hypothesis that supplemental DHA and ARA in infant formula maintains CNS tissue concentrations similar to those that are found for breastfed baboons. We test these hypotheses in both term and preterm baboons at identical post-conceptual ages.

## Methods

### Animals

The Cornell Institutional Animal Care and Use Committee approved the care of animals and the Association for Assessment and Accreditation of Laboratory Animal Care (AAALAC) approved the facility. Female pregnant baboons *(Papio cynocephalu*s) obtained from the Southwest Foundation for Biomedical Research (San Antonio, TX) were used in this study. After confirmation of pregnancy, 29 pregnant baboons were transported to Cornell University (Ithaca, NY). The animals were involved in a series of studies reported recently [21-24] and involving only manipulation of dietary LCP and/or prematurity, as presented here. A complete veterinary examination was performed upon arrival in Ithaca. Pregnant baboons were housed in individual cages in sight of at least one other baboon.

Breastfed neonates were housed with their mothers in a controlled-access nursery where the temperature and humidity were 28°C and 50% respectively, and with a 14 h light and 10 h dark cycle, and consumed breastmilk exclusively during the study period. All other neonates, the "bottle-fed" groups, were removed and placed in a nursery and consumed commercially available infant formula without (minus, "-") or supplemented with (plus, "+") long chain polyunsaturates (LCP).

### Study design and diets

**Figure 1:**
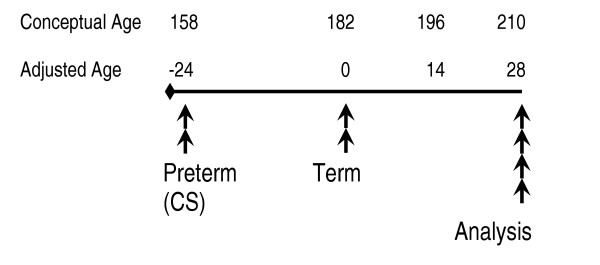
Study timeline for the formula groups. Time is represented as days from conception (conceptual age) or adjusted to time of normal term birth. The preterm (P) groups were taken at -24 days of gestational age by Cesarean section, while the term (T) groups were born spontaneously. All groups were euthanized at 28 days adjusted age, including the breastfed group (B).

A diagram showing the timeline for CS and for euthanasia is presented in Figure 1. All pregnant adult animals consumed a commercial chow, yams, and fruits. The breastfed (B) and term groups (T+ and T-) were delivered vaginally after spontaneous labor and averaged a birthweight of 863 grams, and there were no significant differences among groups. Further details are presented in Table 1.

**Table 1:** Characteristics of baboon neonates.

	Breastfed B	Term, no LCP T-	Term, LCP T+	Preterm, no LCP P-	Preterm, LCP P+
Gender	2F, 3M	5F, 2M	2F, 5M	3F, 2M	3F, 2M
Gestational age at CS^a ^(d)	Spontaneous Labor	Spontaneous labor	Spontaneous labor	156 ± 5	154 ± 2
Age at euthanasia (d)^b^	28	28	28.6	208 ± 2	208 ± 2
Birth weight (g)^c^	875 ± 65^A^	821 ± 119^A^	894 ± 96^A^	632 ± 61^B^	608 ± 71^B^
Body weight at euthanasia (g)^d^	1152 ± 177	1147 ± 113	1107 ± 145	1071 ± 187	1035 ± 220
Brain weight at euthanasia (g)^d^	103 ± 4	98 ± 6	100 ± 11	93 ± 8	94 ± 9

Data are expressed as mean ± SD.

^a^CS, Cesarean section.

^b^Reported as birth age for B and T groups and conceptual (gestational) age for the P groups. The normal gestational period for baboons is 180–182 days. Conceptual age for B and T at euthanasia was within 2 days of that for P+ and P-. Two animals in the T- group and one in the B group were 42 days old at euthanasia. Other than these, all animals were about 208 days post-conception at euthanasia.

^c^Values that do not share common capital letter superscripts are significantly different (*p <*0.05).

^d^No significant differences among groups.

The concentrations of selected LCP in diets are presented in Table 2, and are available in detail in other publications [22,24]. Pregnant and, for the breastfed group, lactating, females consumed a conventional commercial primate diet for the entirety of the study. Normal primate meal contains fish meal as a source of protein, which also adds n-3 long chain polyunsaturated fatty acids (LCP). The breastmilk of these baboons, sampled at four weeks postpartum, contained about 0.68 ± 0.22% DHA, which falls at the higher end of the range of fish-eating human populations [25]. It also contained 20:5n-3 (0.34 ± 0.13%) and 22:5n-3 (0.51 ± 15%) as outlined in more detail elsewhere [22].

**Table 2:** Content of selected LCP in baboon breastmilk and in formulas, as % (w/w).

	Breastmilk	T-,P-	T+	P+
DHA	0.68 ± 0.22	ND	0.30	0.61 ± 0.03
20:5n-3+22:5n-3	0.84 ± 0.16	ND	0.10	0.18 ± 0.10
ARA	0.62 ± 0.12	ND	0.55	1.21 ± 0.09

ND: not detected

The formulas with no LCP were identical for the term (T-) and preterm (P-) groups. The term group that was supplemented with LCP (T+) consumed formula fortified with 0.3% energy DHA and 0.6% energy ARA, as presented in detail previously [24]. For four of these animals, ARA was a component of phospholipid, while for three it was a component of triglyceride. Otherwise, diets were balanced and similar to commercial infant formula [24].

Preterm groups (P+ and P-) were delivered by Cesarian section (CS) at about 24 days preterm, with details also presented in Table 1. Preterm animals supplemented with LCP (P+) consumed formula fortified with 0.6% en DHA and 1.2% en ARA added as an encapsulated powder, and kindly provided by Mead-Johnson Nutritionals [22]. DHA and ARA in these diets were about twice those in the T+ diet, in an attempt to match the DHA content of breastmilk while holding the DHA/ARA ratio constant at about 2:1.

Neonatal tissue was collected at about 28 days adjusted age. This time point was chosen because previous data showed significant neurobehavioral effects within the first few weeks in term rhesus neonates [26]. The neonates were weighed, ketamine was injected intramuscularly, halothane general anesthesia was induced, and euthanasia was performed by exsanguination under continued halothane anesthesia. The brain and spinal cord were removed, wrapped in foil, frozen in liquid nitrogen and stored at -80°C until analysis. A transection between the brain and the spinal cord was made at the upper C1 level, that is, between the first cervical spine and the skull base. The brain weight listed in Table 1 is thus the sum of the brain, cerebellum and the upper part of the cervical spinal cord.

With the exception of cortical regions, CNS structures were dissected and analyzed in their entirety. For cortex, the top 3 mm of gray matter was carefully dissected from the underlying white matter.

### Lipid extraction and analysis

Total lipids were extracted from tissue homogenates by methods reported previously [27]. Fatty acid methyl esters (FAME) were prepared using 14% BF3 in methanol (Sigma Chemical, St. Louis, Mo.). Butylated hydroxytoluene (BHT) was added to solvents as an antioxidant and a known quantity of freshly prepared heptadecanoic acid (99+%, Sigma Chemical, St Louis, MO) in chloroform was added as an internal standard to tissue samples just before extraction. FAME were dissolved in heptane with BHT and stored at -20°C until analysis.

FAME were analyzed using a Hewlett Packard 5890 series II gas chromatography with a BPX 70 column (60 m × 0.32 mm I.D. × 0.25 μm film; SGE, Austin, Tx) and H2 as carrier gas. Quantitative profiles were calculated using methyl-17:0 as an internal standard and an equal weight FAME mixture (68A; Nuchek Prep, Elysian, Mn) to derive response factors for each FAME. Chromatography conditions and calibration details have been reported previously [27]. The DHA and ARA concentrations are expressed as weight percentages of total fatty acids from 14 to 24 carbons.

### Statistics

DHA data are presented as means ± SD. Weight percents of DHA in various CNS regions were tested for significant differences by Duncan's multiple range test with significance declared at p < 0.05. For treatment effects, the Fisher's least significant difference (Fisher's LSD) procedure was used. A one-way analysis of variance (Anova) was performed to test for equivalence of treatment means for either DHA or ARA in a specific CNS region. When significant at the p < 0.05 level, a t-test was performed on a pairwise basis. Statistics were calculated using functions provided in Excel 2000 for WinXP (Microsoft, Redmond, WA). Duncan's multiple range test was performed to compare the relative concentrations of DHA and ARA among CNS regions.

## Results

### CNS DHA concentrations

**Figure 2:**
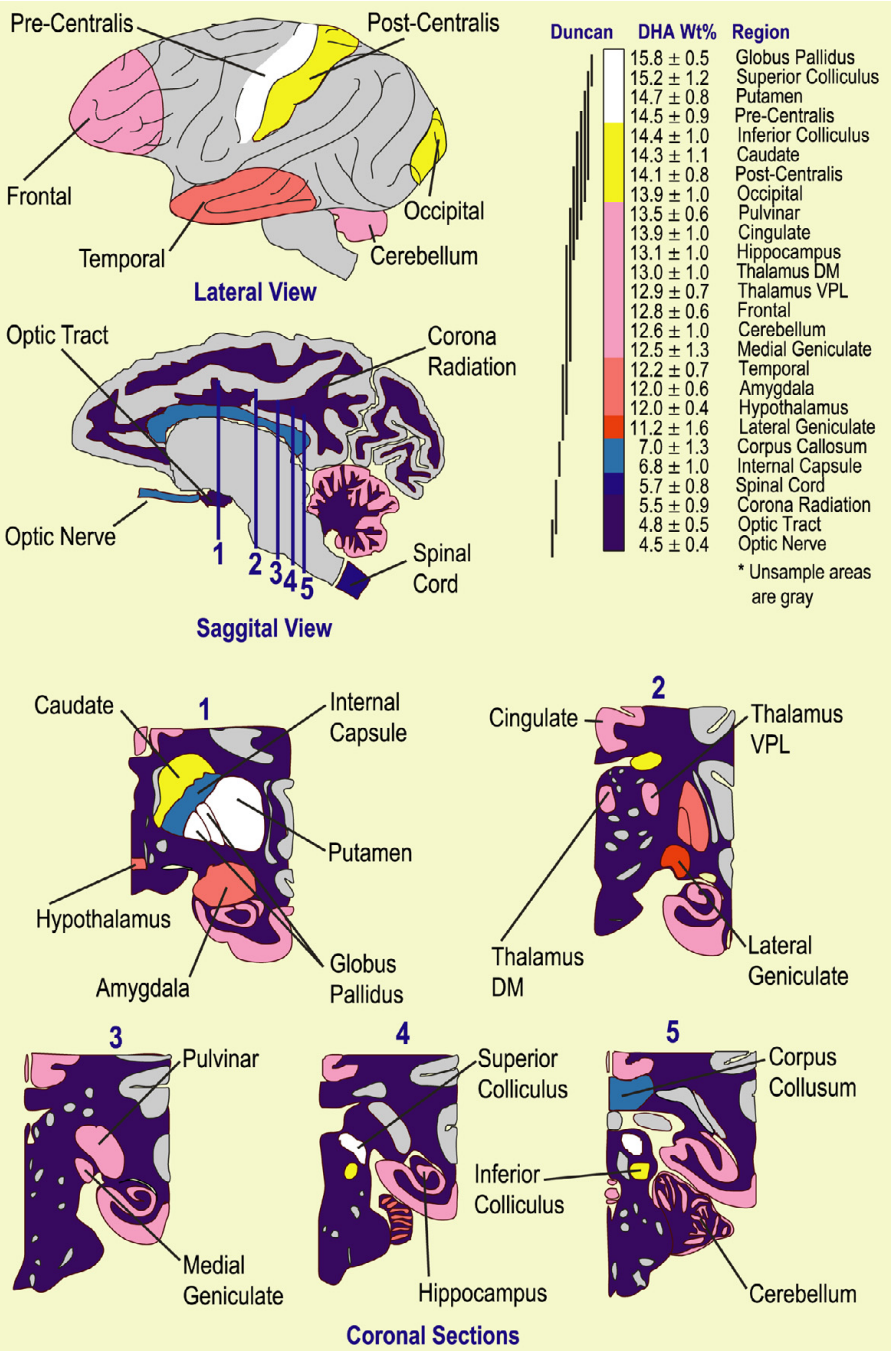
*Baboon Neonate CNS DHA Map*. Schematic diagram of four-week old baboon central nervous system with DHA concentrations color coded and ranked highest (white) to lowest (dark blue). Numbered lines in the parasagittal section refer to coronal sections; in this view, right side is shown with most of the right hemisphere removed for clarity. "Duncan" refers to Duncan's multiple range test; means sharing a line are not statistically different (p < 0.05). The colors each span 10% of the DHA concentration range.

Figure 2 is a schematic presentation of DHA concentrations found in four week old baboon neonate CNS. The coloring reflects DHA concentrations running from white for highest to dark blue for lowest, as shown in the legend; regions colored gray were not analyzed. The globus pallidus has the highest DHA concentration at 15.8 ± 0.5% (w/w), while the optic nerve is lowest at 4.5 ± 0.4%. The legend presents the results of Duncan's multiple range test; regional DHA means that are not significantly different share a common line.

Gray matter DHA was statistically different among cerebral cortex regions and averaged about 14%. This average is greater than we reported elsewhere for cortices of similar baboons [27]. In previous studies, cerebrum was sampled with an indeterminate contribution of white matter. In the present study, about 3 mm of gray matter was carefully removed from the underlying white corona radiation. Our results show that white matter is much poorer in DHA than gray matter. The precentralis gray matter is significantly richer in DHA (14.6 ± 0.9%) than the other cortex lobes, while the temporal has the least DHA (12.2 ± 0.7%).

The distribution of DHA in five coronal sections is presented in an inset of Figure 2. Half of the central regions of the brainstem and surrounding area are presented. Coronal section 1 shows the globus pallidus, putamen and caudate to have very high DHA concentrations (>14.3%), while the surrounding white matter of the coronal radiation is relatively low in DHA (5.5 ± 0.9%). The corona radiation was sampled in regions with only nerve fibers and devoid of neuronal soma, thus nuclei are represented in Figure 2 in gray since they were not sampled. Coronal sections 2–4 present views of the cingulate gyrus, thalamus nuclei, geniculate bodies, pulvinar, colliculi and hippocampus, all rich in DHA. Coronal section 5 shows the white matter of the corpus callosum and coronal radiation, both low in DHA.

Included in Figure 2 is a scale showing DHA values ranked from highest to lowest and statistical differences. Close examination shows that differences in DHA concentrations are relatively small in consecutively ranked regions, 0.6% or less, except for the discontinuity between the lateral geniculate (11.2 ± 1.6%) and corpus callosum (7.0 ± 1.3%). This dichotomy represents a demarcation between predominantly gray or white matter, the latter of which, as previously noted, is relatively poor in DHA.

### CNS ARA concentrations

**Figure 3:**
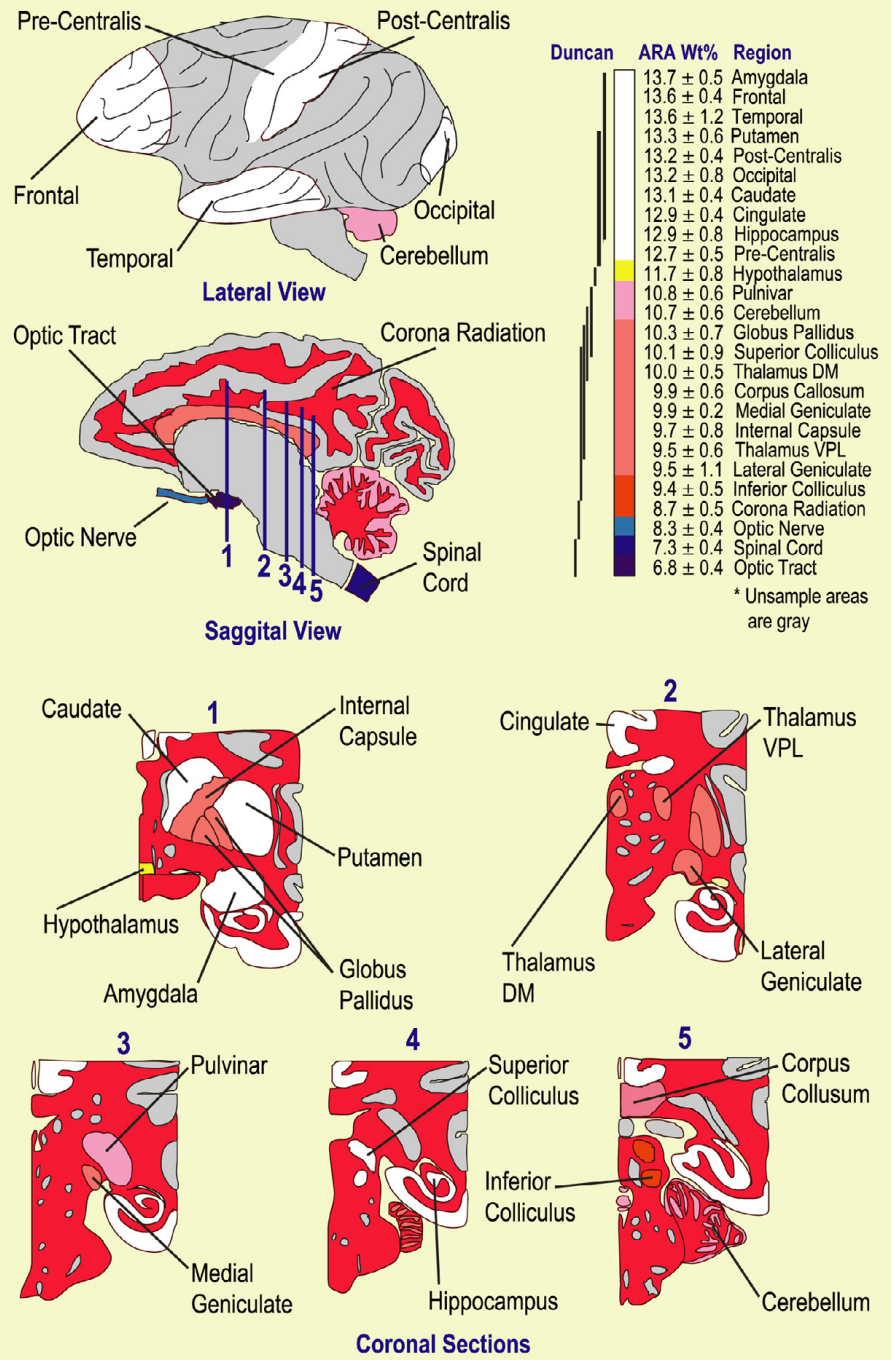
*Baboon Neonate CNS ARA Map*. Schematic diagram of four-week old baboon central nervous system with ARA concentrations color coded and ranked highest (white) to lowest (dark blue). See Figure 2 for key.

Figure 3 is a map of arachidonic acid concentrations in the same CNS regions as presented in figure 2, and was obtained from the same analyses. ARA concentrations range from a high of 13.7 ± 0.5% in the amygdala to a low of 6.8 ± 0.4% in the optic tract, to yield a range of about 2-fold in concentration compared to 3.5-fold for DHA. As with DHA, gray matter is generally richer in ARA than is white matter; however, in contrast to DHA, there is no discontinuity between gray and white matter ARA concentrations.

### Influence of treatments on CNS DHA and ARA

The results of analyses of DHA and ARA concentrations as a function of treatments are presented in tables 3 and 4, respectively. For DHA, one way ANOVA indicated that 23 of the 26 regions showed treatment effects, while only 8 of the 26 regions showed significant ARA treatment effects.

**Table 3:** CNS DHA concentrations (mean ± SD, % w/w) for each treatment. One way Anova was significant (p < 0.05) for all structures except the spinal cord, internal capsule and medial geniculate. The right column shows the results of t tests. Gray matter falls into two classes: 1) LCP supplementation does not support DHA levels found for breastfed animals, detected for cerebral cortex and cerebellum; 2) LCP supplementation does support DHA levels, found in most other gray tissue (basal ganglia, limbic regions, thalamus and midbrain).

CNS Region	Group					Statistics
	B	T+	T-	P+	P-	
Gray,Class 1						
Pre-Centralis	14.4 ± 0.9	12.6 ± 0.6	11.7 ± 1.0	13.0 ± 0.6	10.4 ± 0.7	[**B>all**; T+>P-; P+>T-,P-; T->P-]
Post-Centralis	14.1 ± 0.8	12.7 ± 0.8	11.7 ± 1.0	13.4 ± 0.6	10.4 ± 0.2	[**B>T+,T-,P-**; T+>T-,P-; P+>T-,P-; T->P-]
Occipital	13.9 ± 1.0	11.7 ± 0.9	10.7 ± 0.8	11.8 ± 0.1	8.7 ± 0.6	[**B>all**; T+>P+,P-; T->P+,P-; P+>P-]
Cingulate	13.4 ± 0.9	11.4 ± 0.7	10.7 ± 0.8	11.8 ± 0.8	9.5 ± 0.9	[**B>all**; T+>P-; P+>T-,P-; T->P-]
Frontal	12.7 ± 0.6	11.2 ± 0.8	10.6 ± 0.7	11.4 ± 0.4	8.4 ± 1.0	[**B>all**; T+>P+,P-; T->P-; P+>P-]
Temporal	12.2 ± 0.6	10.5 ± 1.1	10.3 ± 0.8	11.2 ± 0.5	9.0 ± 0.7	[**B>all**; T+,T-,P+>P-]
Cerebellum	12.6 ± 0.9	11.2 ± 0.7	10.6 ± 0.8	11.4 ± 0.2	7.7 ± 0.6	[**B>all**; T+>P-; T-,P+>P-]
Gray, Class 2						
Globus Pallidus	15.8 ± 0.5	15.4 ± 0.6	12.7 ± 2.3	16.6 ± 0.3	13.4 ± 1.5	[**B>T-,P-**; T+>T-,P-; P+>T-,P-]
Putamen	14.7 ± 0.8	13.9 ± 0.9	13.5 ± 0.6	13.6 ± 1.3	11.6 ± 0.8	[**B>T-,P-**; T+,T-,P+>P-]
Caudate	14.3 ± 1.1	13.4 ± 0.8	13.0 ± 1.1	13.5 ± 0.8	11.3 ± 0.4	[**B>T-,P-**; T+,T-,P+>P-]
Superior Colliculus	15.2 ± 1.2	14.2 ± 1.1	13.6 ± 0.5	15.0 ± 0.4	13.4 ± 0.7	[**B>T-,P-**; P+>T-,P-]
Inferior Colliculus	14.4 ± 1.0	12.9 ± 1.4	13.4 ± 1.3	15.0 ± 0.6	13.4 ± 0.4	[**B>T+**; P+>T+,T-,P-]
Amygdala	12.0 ± 0.6	11.4 ± 0.6	10.8 ± 0.9	11.7 ± 0.5	9.4 ± 0.4	[**B>T-,P-**; T+,T-,P+>P-; P+>T-]
Hippocampus	13.0 ± 0.8	11.6 ± 0.5	10.6 ± 1.5	12.6 ± 0.5	9.0 ± 0.5	[**B>T+,T-,P-**; T+,T-,P+>P-; P+>T-]
Hypothalamus	12.0 ± 0.4	11.3 ± 0.6	11.0 ± 0.7	12.0 ± 0.8	10.6 ± 0.4	[**B>T-,P-**; T+>P-; P+>T-,P-]
Pulvinar	13.5 ± 0.6	12.7 ± 0.7	12.5 ± 1.0	14.6 ± 1.1	12.0 ± 0.4	[**B>T-,P-**; P+>all]
Thalamus DM	13.0 ± 1.0	12.4 ± 1.2	12.2 ± 0.9	13.5 ± 0.8	10.9 ± 1.3	[**B>P-**; T+,T-,P+>P-]
Thalamus VPL	12.9 ± 0.7	12.6 ± 0.9	12.2 ± 0.9	13.1 ± 1.7	10.3 ± 1.6	[**B>P-**; T+,T-,P+>P-]
Medial Geniculate	12.5 ± 1.3	12.8 ± 1.1	12.2 ± 0.9	12.6 ± 1.3	11.7 ± 0.8	
Lateral Geniculate	11.2 ± 1.6	10.6 ± 0.9	9.5 ± 0.8	11.7 ± 0.7	8.8 ± 1.8	[**B>T-,P-**; T+>P-; P+>T-,P-]
						
White Matter						
Corpus Callosum	7.0 ± 1.3	7.9 ± 0.7	6.4 ± 0.9	8.2 ± 0.7	6.3 ± 0.7	[T+>T-,P-; P+>B,T-,P-]
Internal Capsule	6.8 ± 1.0	7.0 ± 1.5	6.4 ± 0.7	7.9 ± 0.9	6.3 ± 1.4	
Spinal Cord^1^	5.7 ± 0.3	6.0 ± 1.0	5.6 ± 0.5	6.2 ± 0.5	5.1 ± 0.6	
Corona Radiation	5.5 ± 0.9	5.2 ± 0.9	4.4 ± 0.4	5.7 ± 0.8	4.7 ± 0.7	[B>T-; P+>T-,P-]
Optic Tract	4.8 ± 0.5	4.5 ± 0.4	4.3 ± 0.6	5.1 ± 0.3	3.9 ± 0.4	[B,T+>P-; P+>T+,T-,P-]
Optic Nerve	4.5 ± 0.5	4.0 ± 0.9	3.9 ± 0.4	4.8 ± 0.9	3.6 ± 0.3	[B>T-,P-; T+>P-; P+>all]

^1^Spinal cord is a mixture of white and gray matter.

**Table 4:** CNS ARA concentrations (mean ± SD, % w/w) for eight structures for which a one-way analysis of variance indicated at least one unequal mean (p < 0.05). Anova was not significant for the other 18 structures.

CNS Region	Group					Statistics
	B	T+	T-	P+	P-	

Occipital	13.2 ± 0.8	13.3 ± 0.7	13.6 ± 0.8	14.0 ± 0.5	14.3 ± 0.6	[P->B,T+]
Hypothalamus	11.7 ± 0.7	12.2 ± 0.6	11.4 ± 0.4	12.6 ± 0.7	12.9 ± 0.5	[T+,P+,P->T-; P+,P->B]
Superior Colliculus	10.1 ± 0.9	11.0 ± 0.8	10.6 ± 0.7	11.0 ± 0.6	11.7 ± 0.6	[T+,P+,P->B; P->T-]
Thalamus DM	10.0 ± 0.5	10.3 ± 0.6	9.6 ± 0.8	10.8 ± 0.6	10.7 ± 0.7	[T+,P+,P->T-; P+>B]
Corpus Collosum	9.9 ± 0.6	11.2 ± 0.5	10.2 ± 0.7	11.3 ± 0.5	11.4 ± 0.9	[T+,P+,P->B; T+,P+,P->T-]
Lateral Geniculate	9.5 ± 1.1	10.6 ± 0.6	9.9 ± 0.4	10.7 ± 0.5	10.5 ± 0.4	[T+,P+,P->B; P+>T-]
Inferior Colliculus	9.3 ± 0.5	10.5 ± 1.0	9.3 ± 0.7	10.3 ± 0.5	10.3 ± 0.4	[T+,P+,P->B,T-]
Optic Tract	6.8 ± 0.3	6.9 ± 0.4	7.2 ± 0.7	7.4 ± 0.3	7.7 ± 0.4	[P-,P+>B; P->T+]

We consider the randomized, breastfed control group (B) to be a gold standard against which the others are compared. With the exception of one region, the B group DHA concentration was always among the greatest, while the P- group was always among the lowest. More careful examination of the DHA statistical analysis reveals that the responses of DHA concentrations among gray matter regions fall into two distinct classes, as arranged in Table 3. For all lobes of the cerebral cortex, and for the cerebellum, supplementation with 0.3% (T+) or 0.6% (P+) DHA did not support tissue DHA concentrations similar to those of the breastfed group. This is indicated by the observation that the B group had significantly greater DHA concentration than the other groups, denoted in the table by "B>all". All other areas of gray matter investigated, including the basal ganglia, limbic regions, thalamus and midbrain, fall in a class 2, in which supplemental dietary DHA did restore DHA concentrations to breastfed levels. This is denoted in the table by "B>T-,P-", indicating that the B group DHA is not significantly different from the T+ and P+ group DHA. All the regions listed under class 2 have this pattern, and for two regions, inferior colliculus and hippocampus, the B group DHA is significantly greater than a supplemented group (T+). Another dominant trend among gray regions is significantly greater DHA concentrations in supplemented groups (T+, P+) compared to unsupplemented groups (T-, P-). We can conclude from these data that in gray matter, DHA supplementation increases DHA concentrations in most regions; with the critically important exceptions of the cerebral cortex and cerebellum, supplemental dietary DHA restores CNS DHA concentrations to breastfed levels.

Trends in white matter are not as consistent as the two classes of gray matter. Two of the six white matter regions investigated did not respond to treatments (internal capsule and spinal cord [includes white matter]). The B group DHA was significantly greater than the others in three regions but, uniquely, less than a supplemented group (P+) in the corpus callosum.

The trends for ARA are very different from those for DHA. As noted, treatments were ineffective at altering ARA concentrations in 18 regions. Table 4 shows the eight CNS regions that were significantly influenced by treatments. Remarkably, the B group is of lower ARA concentration than the P- group in every case, and this is significant in 7 of 8 regions. In every region, the P- group is among the set of highest ARA concentration, and B is among the lowest.

For both DHA and ARA, the effects of prematurity *per se *are not strong. For DHA, the T+ group is significantly greater than the P+ group in some cases, and similarly for the T-/P- comparison. Because the groups were adjusted age-matched, they were on formula for different periods: the term groups were on formula for 4 weeks, while the preterm groups were on formula for 7.5 weeks. For this reason alone it is reasonable to expect that the P- group would have lower DHA than the T- group. It should be borne in mind that the supplementation for P+ was twice that for T+, which partially compensates for this difference in exposure to formula DHA. However, in every case, the P+ DHA concentration exceeds that of the T- DHA concentration, showing that the mild prematurity studied here does not severely impair DHA accretion compared to an unsupplemented term group. In other words, DHA concentrations are greater in supplemented preterms than they are in unsupplemented terms, indicating that prematurity itself does not impair DHA accretion.

Table 5 presents results for four other fatty acids of interest in three selected CNS regions (occipital cortex, hippocampus, corpus callosum). Neither 18:0 (stearic acid) nor 22:4n-6 (adrenic acid) showed any statistically significant variation as a function of treatment in these regions; thus, we report means and standard deviations pooled for all treatments. Oleic acid (18:1n-9) was significantly different in two regions but the difference in means was not large, thus we also report pooled means. Importantly, 22:5n-6 (Osbond acid) was significantly elevated in the CNS regions of the groups that did not have dietary DHA, P- and T-. In 25 of 26 CNS regions (except thalamus DM), P- was significantly elevated, while T- was significantly elevated in 22 of 26 regions. Osbond acid generally rises when DHA falls and it is considered a marker of DHA insufficiency [22]. This fatty acid has been previously reported to increase dramatically in n-3 fatty acid deficiency [28] and to fall when deficient animals are supplied with dietary DHA [29]. These data extend our previous report [22] that 22:5n-6 levels rise throughout the CNS when DHA is supplied in the diet with 18:3n-3 levels that are considered to supply sufficient n-3 fatty acids for growth and development.

**Table 5:** Concentrations (mean ± SD) of selected fatty acids in three representative CNS regions. 18:0 (stearic acid) and 22:4n-6 (adrenic acid) were not significantly altered by treatments in these CNS regions or in most others. While 18:1n-9 (oleic acid) was significantly different in occipital cortex and hippocampus but not corpus callosum, the magnitude of the differences was small and overall means are reported. 22:5n-6 (Osbond acid) was significantly elevated in all regions (except thalamus DM), for the P- and T- groups, similar to the patterns shown for the regions in this table.

	Occipital	Corpus Callosum	Hippocampus			
18:0^a^	19.5 ± 0.23	18.4 ± 0.72	19.3 ± 0.40			
18:1n-9	10.6 ± 0.30^b1^	15.9 ± 0.80	11.5 ± 0.30^b2^			
22:4n-6^c^	5.60 ± 0.25	7.060 ± 0.29	5.89 ± 0.19			
22:5n-6	B	T+	T-	P+	P-	

Occipital	1.97 ± 0.23	2.26 ± 0.32	2.47 ± 0.40	1.85 ± 0.23	2.39 ± 0.16	T-,P->B; T+,T-,P->P+
Corpus Callosum	1.16 ± 0.22	1.79 ± 0.26	1.43 ± 0.27	1.41 ± 0.22	1.94 ± 0.33	T+,P->B; T->T+; T+>P+; P->T-,P+
Hippocampus	1.86 ± 0.30	2.27 ± 0.40	2.28 ± 0.41	1.88 ± 0.16	2.33 ± 0.14	T+,T-,P->B; T+,T-,P->P+

^a,c^No significant differences among groups (B, T+, T-, P+, P-).

^b1^Significant group differences: B<T-,P+

^b2^Significant group differences: B<T-,P+, P-

## Discussion

Our fatty acid data were obtained from macroscopically dissectible tissue and therefore contain a mix of cell types and constituents that differ from region to region. Bourre reported the DHA contents of cellular brain fractions of soya oil fed 15-day-old rats [30] as neurons (8.2%), astrocytes (10.6%), synaptosomes (8.5%), oligodendrocyte (5.1%) and myelin (5.8%). These data are qualitatively in accord with our DHA CNS results, considering that gray matter is composed largely of neurons and glia while white matter is composed principally of myelin and oligodendrocytes. However, literature data clearly suggest that DHA concentrations are not explained by a simple mix of cells and cell types. For instance, adult human dorsal-medial (DM) thalamus total cell density and glia/neuron indices are higher than the globus pallidus, though DHA concentrations are lower [31]. Similarly, neuron density (neurons/gram) in humans is in the order occipital lobe > precentralis > frontal lobe > hippocampus, a gradation that also is not consistent with our DHA data [32,33]. Data reported for neuron density of human occipital lobe > temporal lobe > pre-centralis > thalamus were also poorly correlated with our results [34]. Finally, mean neuron volume in humans in the order hippocampus > frontal > precentralis > occipital also failed to show correlation with tissue DHA [32].

A notable trend in the CNS regions richest in DHA is that they are all involved in motor function. The basal ganglia are all integrative centers for motor control and the globus pallidus, caudate and putamen are among the top six in DHA concentration. The cortical region with the highest DHA concentration is the precentralis, which has long been known as the motor cortex. The superior colliculus is a major relay point for saccades, the rapid adjustment of eye position. Of the six regions with the highest DHA, the only region that is not known as a motor region is the inferior colliculus, which plays a role in decoding spatial information in auditory signal processing. Even here a role in motor control system is possible and, notably, no clear function has emerged for the dorsal part of this structure [35].

Recent studies in humans and animals suggest that even a mild DHA deficit may impair motor function. Infant rhesus monkeys on high DHA formula had improved neuromotor scores compared to those on DHA-free diets in the first month of life [26]. Human infants on formula that induced low plasma DHA for the first four months of life scored lower on one year evaluations of motor development than those with better DHA status [36]. Bailey motor development index scores for preterm infants on DHA-containing formula were improved compared to those on formulas with no DHA [37].

Our treatments are a strong test of the hypothesis that LCP supplementation increases CNS LCP concentrations for DHA and ARA, the two LCP that have garnered the most attention for the developing human infant CNS. This is, in part, because the randomized breastfed group is a true gold standard for comparison, and because of the availability of CNS tissue for LCP analysis, neither of which are available in human studies. DHA supplementation at a level matched to that in the breastmilk of these control baboons maintained DHA levels in many but not all CNS regions. The cerebral cortex and cerebellum, both physically large regions requiring proportionately more DHA, were unable to maintain levels similar to controls. We have previously speculated [22] that this may be due to the presence of significant amounts of 20:5n-3 and 22:5n-3 in breastmilk (0.85%), which are thought to be much more efficient precursors of DHA than 18:3n-3 [38], quantitatively the most prominent omega-3 PUFA in breastmilk and formula. Another important consideration is that these regions have developmentally later peak growth profiles than basal and limbic regions, which develop early in gestation [39]. We can speculate that the massive requirement for DHA imposed by aggressive growth during this period renders these regions more vulnerable to DHA insufficiency, and that biosynthesis cannot keep up with demand. We thus accept the hypothesis that supplemental DHA improves CNS tissue status, with the proviso that current levels may be insufficient to maintain levels in cortex. Notably, current formula DHA levels in the USA are 0.15%–0.35% (w/w), which is about 1/4 to 1/2 that used in the P+ group, though the T+ group was similar to commercial formulas with a DHA concentration of about 0.3%. Finally, we note that previous studies of neonatal rhesus monkeys have shown an increase in cortex phosphatidylethanolamine DHA from about 15% at birth to 22% and 34% at 22 months in frontal and occipital cortex, respectively [28]. CNS demands for DHA clearly continue well beyond the perinatal period as the CNS matures.

The influence of our treatments on CNS ARA concentrations is radically different from that for DHA. Most of the CNS regions we investigated are nearly immune to supplementation at levels twice that in current infant formulas (1.2%, w/w, for the P+ group). In fact, the significant trends were, in some respects, opposite to what would have been expected from the simple model that more dietary ARA should result in more CNS ARA. In particular, the breastfed group tended to have the lowest ARA, while the group longest exposed to ARA-free formula, the P- group, tended to have the highest ARA concentrations. Thus, the hypothesis that ARA-supplemented formula always increases CNS ARA is rejected. CNS ARA concentrations are clearly under tighter control than DHA concentrations, a fact that may derive from ARA's role as precursor of potently bioactive eicosanoids. Very recent evidence suggests that DHA is also a precursor of oxygenated derivatives, docosanoids; however the bioactivity of these compounds and their biochemistry are not fully described [13].

## Conclusion

In summary, these data reveal that DHA and ARA concentrations in the CNS are highly region-specific and are unexpectedly high in the deep CNS regions embedded in white matter of much lower DHA and ARA concentration. Supplementation improves DHA concentrations and in all but the cerebral cortex, maintains DHA at levels similar to that of breastfed controls. Prematurity *per se *does not severely impair accretion of DHA. ARA is much less sensitive to dietary manipulation than DHA, and the response of CNS regions to ARA supplementation is complex. Future research targeting functions controlled and mediated by CNS structures other than the cerebrum and visual system that are likely to be sensitive to DHA nutrition is indicated.

## Abbreviations

ARA, arachidonic acid; DHA, docosahexaenoic acid; FAME, fatty acid methyl ester; LCP, long chain polyunsaturated fatty acids; PUFA, polyunsaturated fatty acids.

## Competing interests

JTB is a consultant for Mead-Johnson Nutritionals. All other authors declare that they have no competing interests.

## Authors' contributions

Participation was as follows: Project conception (GYD, PWN, JTB); Study design (GYD, VW, PWN, JTB); Live animal management (GYD, ATH, ESN, VW); Dissection and laboratory analysis (GYD); Interpretation of results (GYD, JTB), manuscript preparation (GYD, ATH, JTB). All authors approved the final manuscript.

## Pre-publication history

The pre-publication history for this paper can be accessed here:


